# Cross-sectional association between hs-CRP/HDL-C ratio and physical frailty among middle-aged and older adults: findings from a population-based study

**DOI:** 10.3389/fpubh.2025.1564206

**Published:** 2025-05-09

**Authors:** Yina Wang, Jing Su, Yang Wang

**Affiliations:** Department of Geriatrics, The Second Xiangya Hospital of Central South University, Changsha, Hunan, China

**Keywords:** frailty, hs-CRP, HDL-C, NHANES, plasma proteins

## Abstract

**Background:**

Frailty, characterized by functional decline and disability, is an emerging public health concern in aging populations. Chronic inflammation and low high-density lipoprotein cholesterol (HDL-C) levels are key contributors to the progression of frailty. This study aims to examine the association between the ratio of high-sensitivity C-reactive protein (hs-CRP) to HDL-C and frailty among middle-aged and older adults in the United States.

**Methods:**

Our study included participants aged 45 years and older from the 2015–2020 National Health and Nutrition Examination Survey (NHANES). Logistic regression and restricted cubic spline (RCS) analysis were utilized to assess the relationship between the hs-CRP/HDL-C ratio and frailty, adjusting for potential confounding covariates. Mediation analysis was performed to determine whether plasma proteins mediated this association. Least absolute shrinkage and selection operator (LASSO) regression was employed to identify variables strongly correlated with frailty, and a nomogram was subsequently developed based on these variables.

**Results:**

Our study included 3,626 middle-aged and older participants, among whom 787(21.7%) were identified as frailty. After adjusting for all covariates, a high hs-CRP/HDL-C ratio was identified as a significant risk factor for frailty (OR = 1.736, 95% CI: 1.009–2.988). RCS analysis disclosed a nonlinear correlation between the hs-CRP/HDL-C ratio and frailty incidence. Furthermore, mediation analysis suggested that albumin and globulin partially mediated this association, accounting for 37.82% and 11.23% of the indirect effect, respectively. A nomogram, constructed using variables selected via LASSO regression, exhibited promising discriminative ability, with an area under the curve (AUC) of 79.7% (95% CI: 77.7–81.75%).

**Conclusion:**

Our findings suggest that a higher hs-CRP/HDL-C ratio is associated with an increased risk of frailty among middle-aged and older adults. Albumin and globulin partially mediate this relationship. Additionally, the nomogram developed in our study shows strong predictive ability for identifying individuals at high risk of frailty in this population.

## 1 Introduction

The global aging population is growing rapidly (1). Frailty, an age-associated clinical syndrome, serves as an important indicator for identifying individuals at high risk of adverse outcomes, including falls, hospitalization, disability, and mortality (2, 3). It is a multifactorial condition influenced by various biological mechanisms such as chronic inflammation, oxidative stress, nutritional status(e.g., albumin and diet), aging, and physical activity (4). The frailty index (FI) is a widely accepted tool for assessing frailty across populations (5). Given the wide range of complications associated with frailty, it is crucial to identify reliable biomarkers to predict and slow the progression of frailty.

Inflammation plays a crucial role in the development of frailty (6, 7). C-reactive protein (CRP), a well-established marker of inflammation, has been linked to reduced muscle mass and strength (8). However, the relationship between CRP and frailty remains inconclusive. In an observational study by Kamil R. J. et al., no significant association was found between CRP levels and frailty risk (9). Conversely, Luo et al. reported elevated CRP levels among individuals with frailty (10). High-sensitivity CRP (hs-CRP) assays, commonly used in clinical practice, enable precise detection of CRP levels and serve as valuable predictors of cardiovascular disease and stroke (11, 12). In addition to inflammatory markers, lipid metabolism also plays a role in frailty. Reduced high-density lipoprotein cholesterol (HDL-C) levels are frequently observed in individuals with chronic conditions (13). HDL-C, known for its anti-inflammatory properties, has been inversely associated with frailty in older adults (14, 15). Both hs-CRP and HDL-C are common biomarkers for assessing inflammation and lipid status in clinical settings. Previous research has established that the hs-CRP/HDL-C ratio may serve as a risk indicator for new stroke and cardiovascular issues (16). However, few studies have examined the relationship between the hs-CRP/HDL-C ratio and frailty in large, population-based cohorts. Our research aims to explore whether the hs-CRP/HDL-C ratio can serve as a useful biomarker for identifying frailty among middle-aged and older adult individuals. To achieve this, we will analyze extensive data from the National Health and Nutrition Examination Survey (NHANES) spanning from 2015 to 2020. This study presents an opportunity to identify a more reliable biomarker for frailty assessment in middle-aged and older populations.

## 2 Methods

### 2.1 Data collection and study population

**Figure 1:**
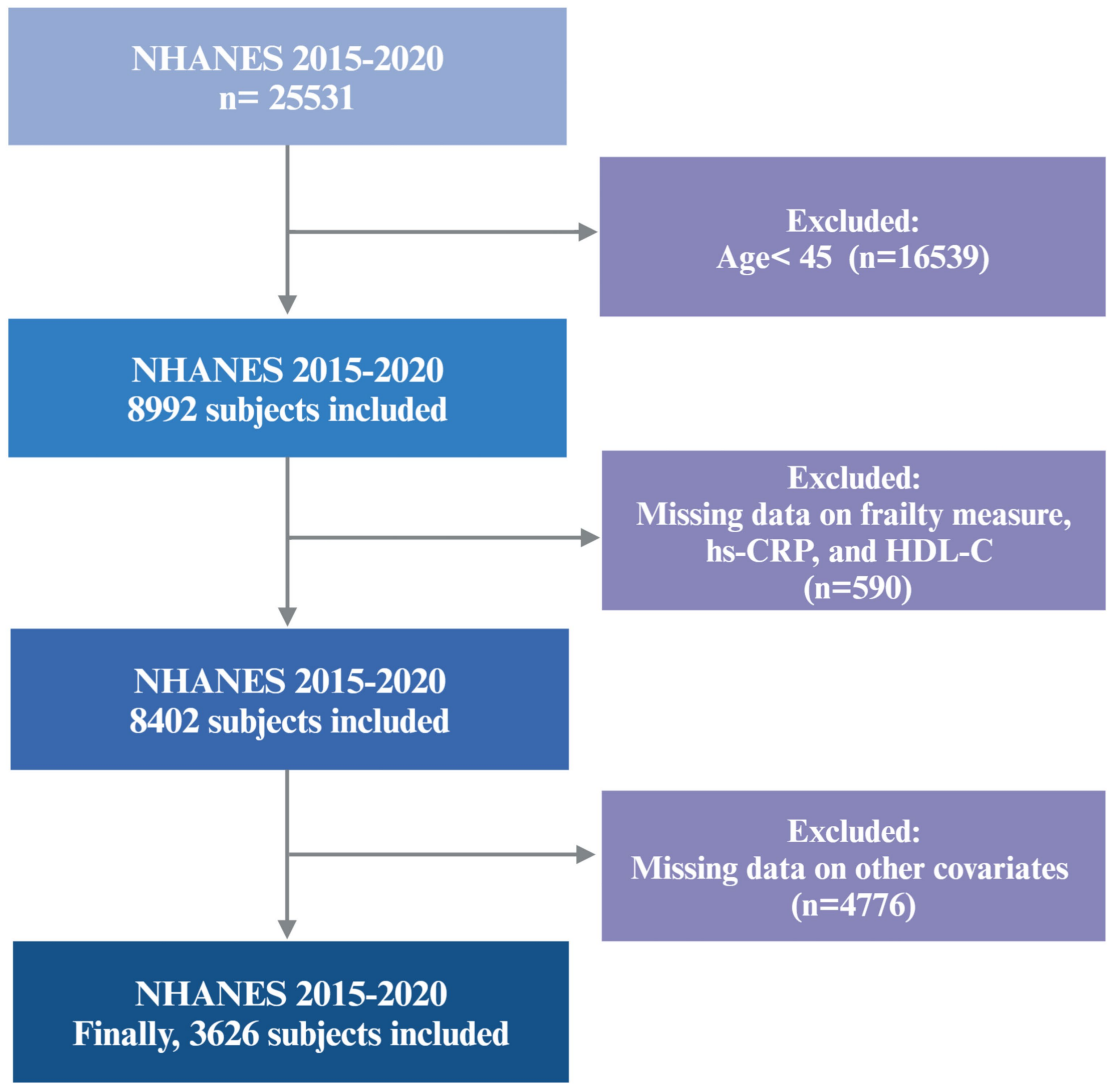
The participant screening flowchart.

The data for this study were obtained from the 2015–2020 cycles of the NHANES, a publicly available cross-sectional survey designed to evaluate the health and nutritional status of the U.S. population. Because of the COVID-19 pandemic, data obtained from 2019 to March 2020 were combined with data from the 2017–2018 cycle to form a nationally representative pre-pandemic dataset covering the period from 2017 to March 2020. A total of 3,626 participants aged 45 years and older were included in the final analysis. Individuals with incomplete data required to construct the frailty index, or with missing data for hs-CRP, HDL-C, or other selected covariates, were excluded. The final cohort is representative of approximately 7.05 million non-institutionalized residents of the U.S. A flowchart detailing participant recruitment is provided in Figure 1.

### 2.2 The hs-CRP/HDL-C ratio

During all three cycles of the NHANES, serum samples were obtained for analysis. Serum levels of hs-CRP (mg/L) and HDL-C (mg/dL) were analyzed at the University of Minnesota, Minneapolis, MN. The hs-CRP/HDL-C ratio was determined by dividing the concentration of hs-CRP by the concentration of HDL-C. The hs-CRP/HDL-C ratio was categorized into quartiles (Q1–Q4) based on its distribution.

### 2.3 Frailty index

Frailty was evaluated using the frailty index (FI), constructed from 49 health deficits as presented in Supplementary Table S1 (17, 18). The health deficits encompassed various aspects such as cognition, dependency, depressive symptoms, comorbidities, hospital utilization and access to care, physical performance, and laboratory values. The FI was computed by dividing the total number of deficits identified in each participant by the overall number of deficits considered. This index yields a score ranging from 0 to 1, with a higher score indicating a higher number of deficits and a frailer condition. In accordance with previous research (19, 20), frailty status was categorized based on the FI score: an FI score of <0.25 denoting non-frailty, and an FI score of ≥0.25 denoting frailty.

### 2.4 Covariates

Multiple sociodemographic factors including age, gender (male, female), marital status (non-single and single), ethnicity (Non-Hispanic White, Non-Hispanic Black, Mexican American, and Other Race), and educational level (> High school, High school, <High school) were collected as covariates. Additionally, the family income-to-poverty ratio (FITPR) was classified as < 1.3, 1.3–3.5, and > 3.5 based on the household income to poverty guidelines ratio (21). Lifestyle factors such as physical activity and smoking status were included. Physical activity was assessed using metabolic equivalent minutes per week, calculated by multiplying the total time spent in transportation, work, and recreational activity. Physical activity was categorized as <500 and ≥500 metabolic equivalent minutes per week, based on the World Health Organization’s recommended physical activity level (22). Smoking status was classified into two categories: “no” (individuals who smoked fewer than 100 cigarettes in their lifetime, or previously smoked more than 100 cigarettes but quit and are currently nonsmokers) and “yes” (individuals who smoked more than 100 cigarettes in their lifetime and are currently smokers) (23).

For energy intake, a 2-day dietary value obtained from Day 1 and Day 2 or using data from Day 1 when Day 2 data were missing. Alcohol consumption was categorized into five groups: former drinkers, nondrinkers, mild drinkers, moderate drinkers, heavy drinkers, as indicated in previous studies (24). Medical condition data encompassed BMI (weight in kg divided by height in meters squared), serum creatinine concentration measured in mg/dL using the Jaffe rate method, and various health statuses. Diabetes mellitus status was classified into four categories: Diabetes mellitus (DM, defined as fasting glucose ≥ 7.0 mmol/L, random plasma glucose ≥ 11.1 mmol/L, two-hour OGTT blood glucose ≥ 11.1 mmoL/L; glycohemoglobin HbA1c ≥ 6.5%, self-reported physician-diagnosed diabetes, or current use of antidiabetic drugs), pre-DM (fasting glucose level should be between 6.11 mmol/L and 7.0 mmol/L, or two-hour OGTT blood glucose range from 7.7 mmol/L to less than 11.1 mmol/L), or ‘no’ (normal glucose levels). Hypertension status was determined as ‘yes’ for elevated blood pressure or medication use, and ‘no’ for normal blood pressure. Similarly, stroke status was classified as either “yes” or “no” based on participants’ self-reported history of physician-diagnosed stroke.

### 2.5 Statistical analysis

Data analysis was conducted using R software version 4.4.0, incorporating the nhanesR (0.9.5.0), ggplot2, rms, and mediation packages (23). In alignment with analytic guidelines, sample weights were incorporated to ensure the national representativeness of the survey. Baseline characteristics of participants were stratified by frailty status, with survey-weighted mean ± standard error [SE] for continuous variables and survey-weighted proportions for categorical variables. Group comparisons were conducted utilizing survey-weighted t-test for continuous variables and survey-weighted Rao-Scott chi-square test for categorical variables. The association between the hs-CRP/HDL-C ratio and frailty status was examined through univariate and multivariate logistic regression models. Covariates were selected based on prior literature and clinical relevance. The crude model was unadjusted for any covariates. Model I adjusted for age, gender, ethnicity, and education status. Model II further included age, gender, marital status, ethnicity, education status, BMI, energy intake, FITPR, physical activity, smoking status, alcohol user status, creatinine levels, and comorbidities (hypertension, DM, and stroke). Results of the logistic regression were presented as odds ratios (ORs) with 95% confidence intervals (CIs). Restricted cubic splines (RCS) regression were used to evaluate potential non-linear relationships between the hs-CRP/HDL-C ratio and FI score. Additionally, multivariable linear regression was used to explore the associations between the hs-CRP/HDL-C ratio and plasma proteins (albumin and globulin). Causal mediation analysis was conducted to investigate if the association between the hs-CRP/HDL-C ratio and frailty status was mediated by plasma proteins. The mediation effect was estimated using bootstrapping with 1,000 resamples, and indirect, direct, and total effects were reported.

To develop and validate a predictive nomogram, the dataset was randomly divided into a training set (70% of the data) and a validation set (30% of the data), stratified by frailty status. Least absolute shrinkage and selection operator (LASSO) regression, implemented using the glmnet package, was applied to identify the key predictors in the training set. The largest penalty parameter lambda (*λ*) within one standard error of the minimum binomial deviation, was selected based on tenfold cross-validation with 1,000 iterations. Variables with non-zero coefficients were retained for the final model. A nomogram was then constructed using multivariate logistic regression, and its discriminatory performance was evaluated using the receiver operating characteristic (ROC) curve. Statistical significance was defined as a two-sided *p* value of less than 0.05.

## 3 Results

### 3.1 Baseline characteristics

Baseline characteristics stratified by frailty status are presented in Table 1. The study comprised 3,626 participants (2,839 non-frailty and 787 frailty), comprising 1935 males and 1,691 females, with a mean age of 59.67 ± 0.34 years. The frailty group had a significantly higher mean age (63.59 ± 0.55 years) compared to the non-frailty group (58.97 ± 0.36 years, *p* < 0.0001). Additionally, the proportion of older individuals (≥75 years) was significantly higher in the frail group (17.07%) compared to the non-frail group (7.58%). Notably, the frailty group displayed higher values in hs-CRP/HDL-C ratios, creatinine levels, and a greater incidence of single status, diabetes mellitus(DM), hypertension, and stroke than the non-frailty group. Moreover, the frailty group displayed less values in energy intake than the non-frailty group. Significant intergroup differences were identified across a range of variables, including age, marital status, ethnicity, education level, BMI, energy intake, FITPR, physical activity, creatinine levels, smoking status, alcohol consumption, hs-CRP/HDL-C ratio, diabetes mellitus status, hypertension status, and stroke status, as delineated in Table 1.

**Table 1:** Baseline characteristics of participants.

Variables	Total	Non-frailty	Frailty	*p*-value
*n* (sample size)	3,626	2,839	787	
*N* (weighted- sample size)	70,478,619	59,879,934	10,598,685	
Age (years)	59.67 ± 0.34	58.97 ± 0.36	63.59 ± 0.55	< 0.0001
Age (*n*, %)				< 0.0001
45–60 years	1729 (53.66)	1,445 (56.38)	284 (38.33)	
60–75 years	1,471 (37.33)	1,113 (36.04)	358 (44.60)	
≥75 years	426 (9.01)	281 (7.58)	145 (17.07)	
Gender (*n*, %)				0.08
Female	1,691 (49.62)	1,304 (48.69)	387 (54.87)	
Male	1935 (50.38)	1,535 (51.31)	400 (45.13)	
Marital Status (*n*, %)				< 0.0001
Non-single	2,293 (71.79)	1893 (74.23)	400 (58.01)	
Single	1,333 (28.21)	946 (25.77)	387 (41.99)	
Ethnicity (*n*, %)				< 0.0001
Non-Hispanic White	1,512 (75.12)	1,190 (76.69)	322 (66.22)	
Non-Hispanic Black	807 (8.27)	590 (7.33)	217 (13.58)	
Mexican American	424 (4.98)	350 (5.07)	74 (4.44)	
Other Race	883 (11.64)	709 (10.91)	174 (15.76)	
Education (*n*, %)				< 0.0001
<High school	606 (8.40)	429 (7.20)	177 (15.20)	
High school	826 (23.08)	609 (21.52)	217 (31.89)	
>High school	2,194 (68.51)	1801 (71.28)	393 (52.91)	
BMI (*n*, %)				< 0.0001
<25	827 (24.15)	718 (26.12)	109 (13.05)	
25–30	1,282 (35.95)	1,032 (36.64)	250 (32.06)	
≥ 30	1,517 (39.90)	1,089 (37.24)	428 (54.89)	
Energy intake (kcal)	2135.93 ± 17.53	2162.94 ± 18.92	1983.34 ± 41.45	< 0.001
FITPR (*n*, %)				< 0.0001
<1.5	1,013 (15.00)	673 (11.76)	340 (33.28)	
1.5–3.37	1,116 (25.87)	867 (24.70)	249 (32.52)	
>3.37	1,497 (59.13)	1,299 (63.54)	198 (34.20)	
Physical activity (*n*, %)				0.04
<500	674 (17.14)	499 (16.28)	175 (21.99)	
≥500	2,952 (82.86)	2,340 (83.72)	612 (78.01)	
Smoke (*n*, %)				< 0.0001
Yes	619 (14.64)	430 (12.88)	189 (24.54)	
No	3,007 (85.36)	2,409 (87.12)	598 (75.46)	
Alcohol user (*n*, %)				< 0.001
Former	308 (8.77)	216 (7.66)	92 (15.08)	
Never	433 (8.69)	357 (9.08)	76 (6.49)	
Mild	1,688 (47.52)	1,338 (48.38)	350 (42.69)	
Moderate	636 (19.89)	494 (20.01)	142 (19.22)	
Heavy	561 (15.12)	434 (14.88)	127 (16.52)	
Creatinine (mg/dL)	78.56 ± 0.63	77.43 ± 0.60	84.94 ± 2.00	< 0.0001
hs-CRP/HDL-C ratio	0.07 ± 0.00	0.07 ± 0.00	0.13 ± 0.01	< 0.0001
hs-CRP/HDL-C ratio (*n*, %)				< 0.0001
Q1	934 (29.73)	810 (32.01)	124 (16.87)	
Q2	872 (23.31)	706 (24.02)	166 (19.34)	
Q3	917 (24.33)	698 (23.77)	219 (27.48)	
Q4	903 (22.63)	625 (20.20)	278 (36.32)	
Hypertension (*n*, %)				< 0.0001
Yes	2073 (50.20)	1,435 (44.83)	638 (80.54)	
No	1,553 (49.80)	1,404 (55.17)	149 (19.46)	
Diabetes mellitus (*n*, %)				< 0.0001
DM	926 (19.44)	550 (14.98)	376 (44.67)	
Pre-DM	367 (11.14)	315 (11.33)	52 (10.07)	
No	2,333 (69.41)	1974 (73.69)	359 (45.25)	
Stroke (*n*, %)				< 0.0001
Yes	193 (3.53)	78 (1.90)	115 (12.75)	
No	3,433 (96.47)	2,761 (98.10)	672 (87.25)	

hs-CRP, high-sensitivity C-reactive protein; HDL-C, high-density lipoprotein cholesterol; DM, Diabetes mellitus; BMI, body mass index; FITPR, family income-to-poverty ratio. Continuous variables are presented as survey-weighted mean ± standard error [SE], and categorical variables are presented as survey-weighted counts and percentages. For continuous variables: *p* values were calculated by survey-weighted t-test. For categorical variables: *p* values were calculated by survey-weighted Chi-square test.

### 3.2 Association between the hs-CRP/HDL-C ratio and frailty status

**Figure 2:**
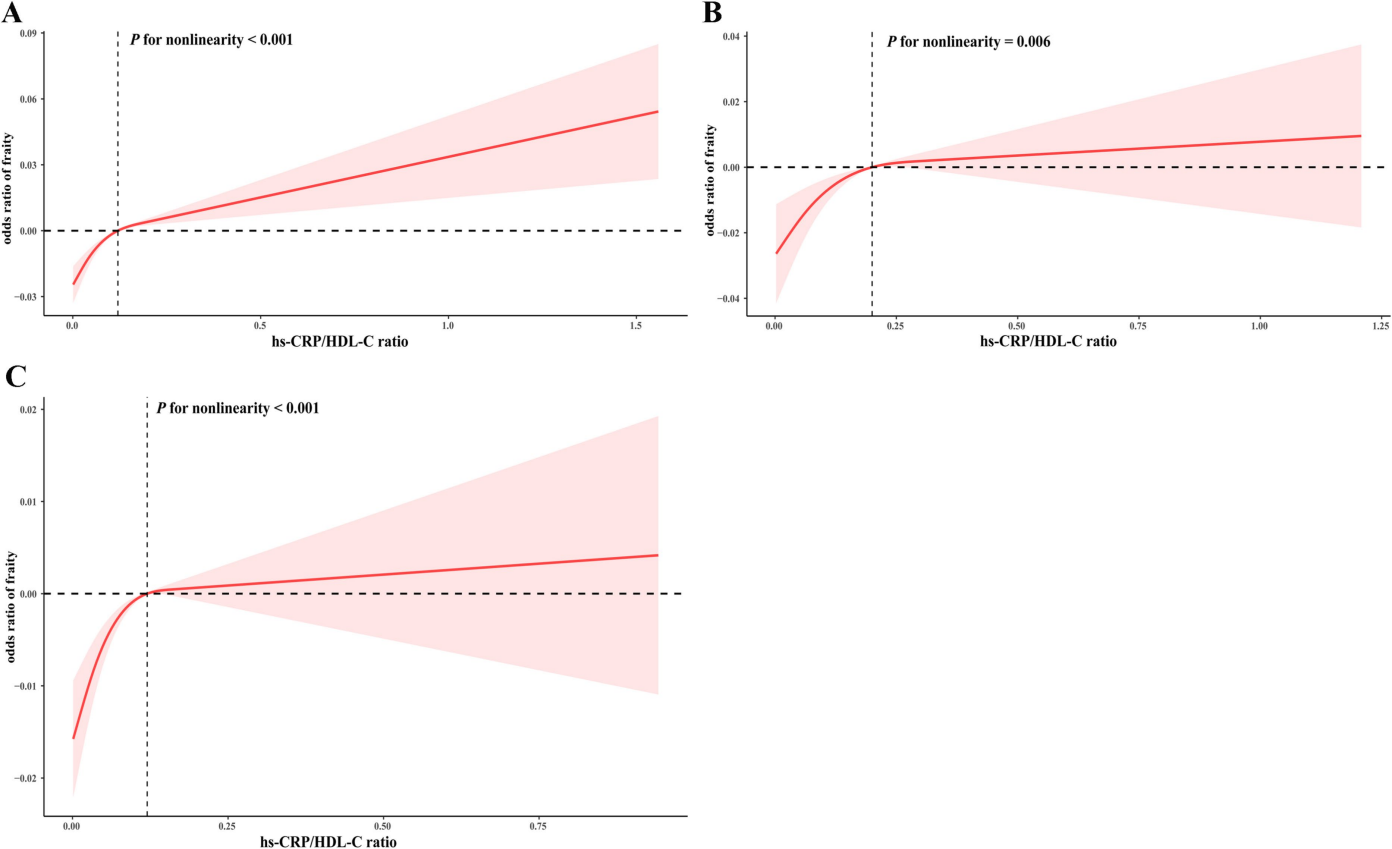
RCS for the correlation between the hs-CRP/HDL-C ratio and frailty index. The RCS analysis was performed in the overall population **(A)**, as well as in subgroups specifically categorized as frailty **(B)** or non-frailty **(C)** individuals.

Table 2 illustrates the associations between the quartiles of the hs-CRP/HDL-C ratio and frailty status. After adjusting for all confounding covariates, individuals in the highest quartile of the hs-CRP/HDL-C ratio displayed a significantly higher odds ratio (OR) for frailty compared to those in the lowest quartile (OR = 1.736, 95% CI: 1.009, 2.988). The multivariable-adjusted RCS analysis revealed a non-linear relationship between the hs-CRP/HDL-C ratio and frailty, observed in the overall population as well as in subgroups of frailty and non-frailty individuals (*P* for nonlinear < 0.05, Figures 2A–C).

**Table 2:** Correlations between the four quartiles of hs-CRP/HDL-C ratio and frailty status.

	Crude model		Model I		Model II	
Variables	Crude OR (95%CI)	*p*-value	Adjusted OR (95%CI)	*p*-value	Adjusted OR (95%CI)	*p*-value
hs-CRP/HDL-C ratio						
Q1 group	Reference		Reference		Reference	
Q2 group	1.528 (1.035, 2.255)	0.034	1.402 (0.947, 2.076)	0.089	1.057 (0.709, 1.575)	0.769
Q3 group	2.194 (1.585, 3.036)	<0.0001	2.126 (1.547, 2.921)	<0.0001	1.232 (0.797, 1.905)	0.320
Q4 group	3.411 (2.364, 4.921)	<0.0001	3.391 (2.322, 4.954)	<0.0001	1.736 (1.009, 2.988)	0.047
*P* for trend		<0.0001		<0.0001		0.037

Crude Model: unadjusted.

Model I: adjusted for age, gender, education status, and ethnicity.

Model II: adjusted for age, gender, marital status, ethnicity, education status, BMI, energy intake, FITPR, physical activity, smoking status, alcohol user status, creatinine levels, hypertension, diabetes mellitus, and stroke status. Bold values indicate statistically significant results (*p* < 0.05).

### 3.3 Associations between the hs-CRP/HDL-C ratio and plasma proteins

Plasma proteins, particularly albumin and globulin, have been associated with nutrition and inflammation (25). In our study, we employed a multivariate linear regression model to explore the connections between the hs-CRP/HDL-C ratio and these plasma proteins. Notably, as depicted in Table 3, the hs-CRP/HDL-C ratio displayed a significantly negative correlation with albumin (*β* = −0.011; 95% CI –0.013, –0.010, *p* < 0.0001), while showing a positive association with globulin (*β* = 0.007; 95% CI 0.006, 0.008, *p* < 0.0001).

**Table 3:** Multivariate linear regression of hs-CRP/HDL-C ratio with plasma proteins.

	*n*	*β*	95%CI	*p*-value
hs-CRP/HDL-C ratio				
albumin (g/L)	3,624	−0.011	(−0.013, −0.010)	<0.0001
globulin (g/L)	3,624	0.007	(0.006, 0.008)	<0.0001

### 3.4 The mediating role of plasma proteins in the association between the hs-CRP/HDL-C ratio and frailty

**Figure 3:**
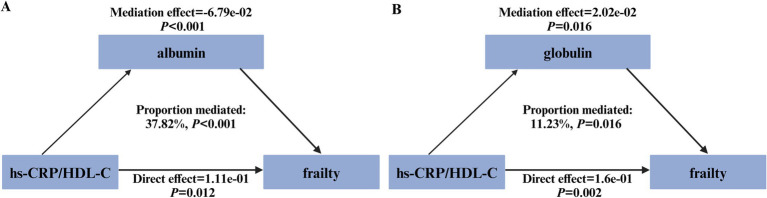
Path diagram illustrating the mediating role of plasma proteins. The graphs in **(A,B)** showed the mediating role of albumin and globulin, respectively.

As illustrated in Figure 3, both albumin and globulin displayed significant mediation effects on the association between the hs-CRP/HDL-C ratio and frailty, with mediated proportions of 37.82% (*p* < 0.001, Figure 3A) and 11.23% (*p* = 0.016, Figure 3B), respectively.

### 3.5 LASSO regression and nomogram model

**Figure 4:**
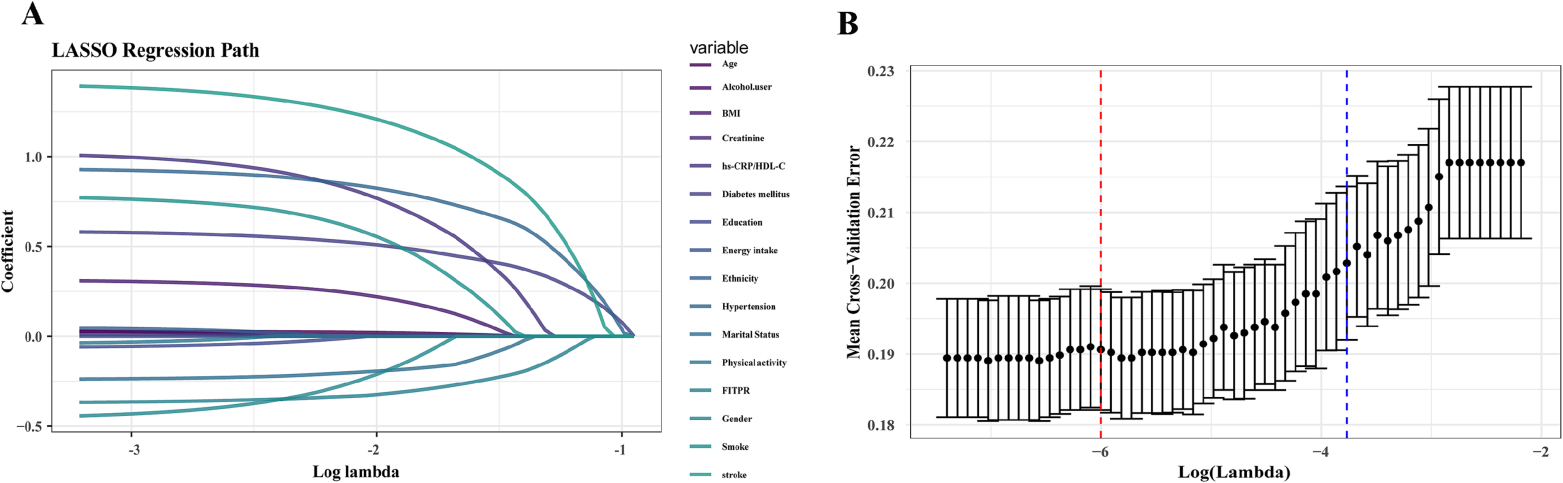
LASSO regression analysis to identify key variables most correlated with frailty. **(A)** A LASSO regression coefficient plot was constructed. Each curve represents the trajectory of an individual feature coefficient. **(B)** Cross-validation plot.

**Figure 5:**
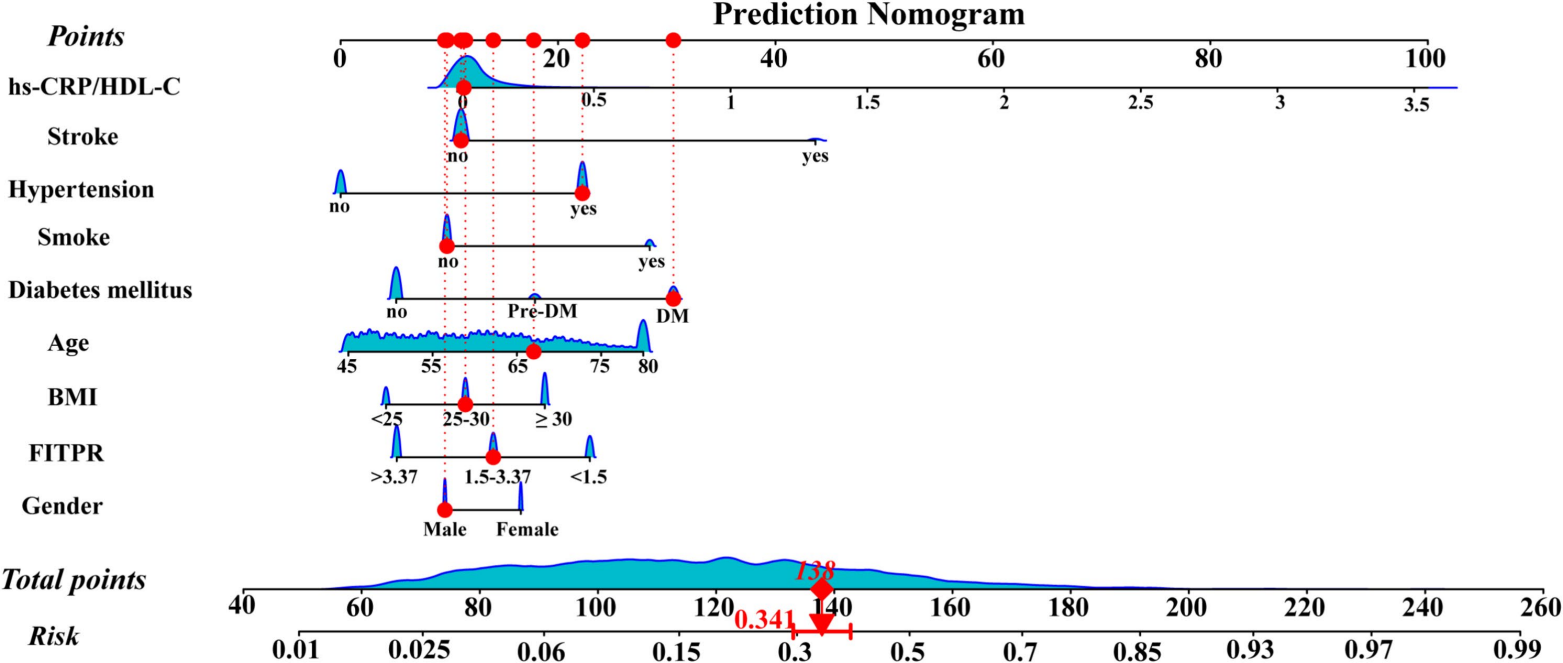
Nomogram for predicting the risk of frailty. The points corresponding to each feature were summed to obtain a total score, and a vertical line was drawn from the total score to determine the ‘frailty risk’. The red points illustrate a specific example: for a 67-year-old male participant with DM and hypertension, but a low hs-CRP/HDL-C ratio, the predicted probability of frailty is 34.1%.

**Figure 6:**
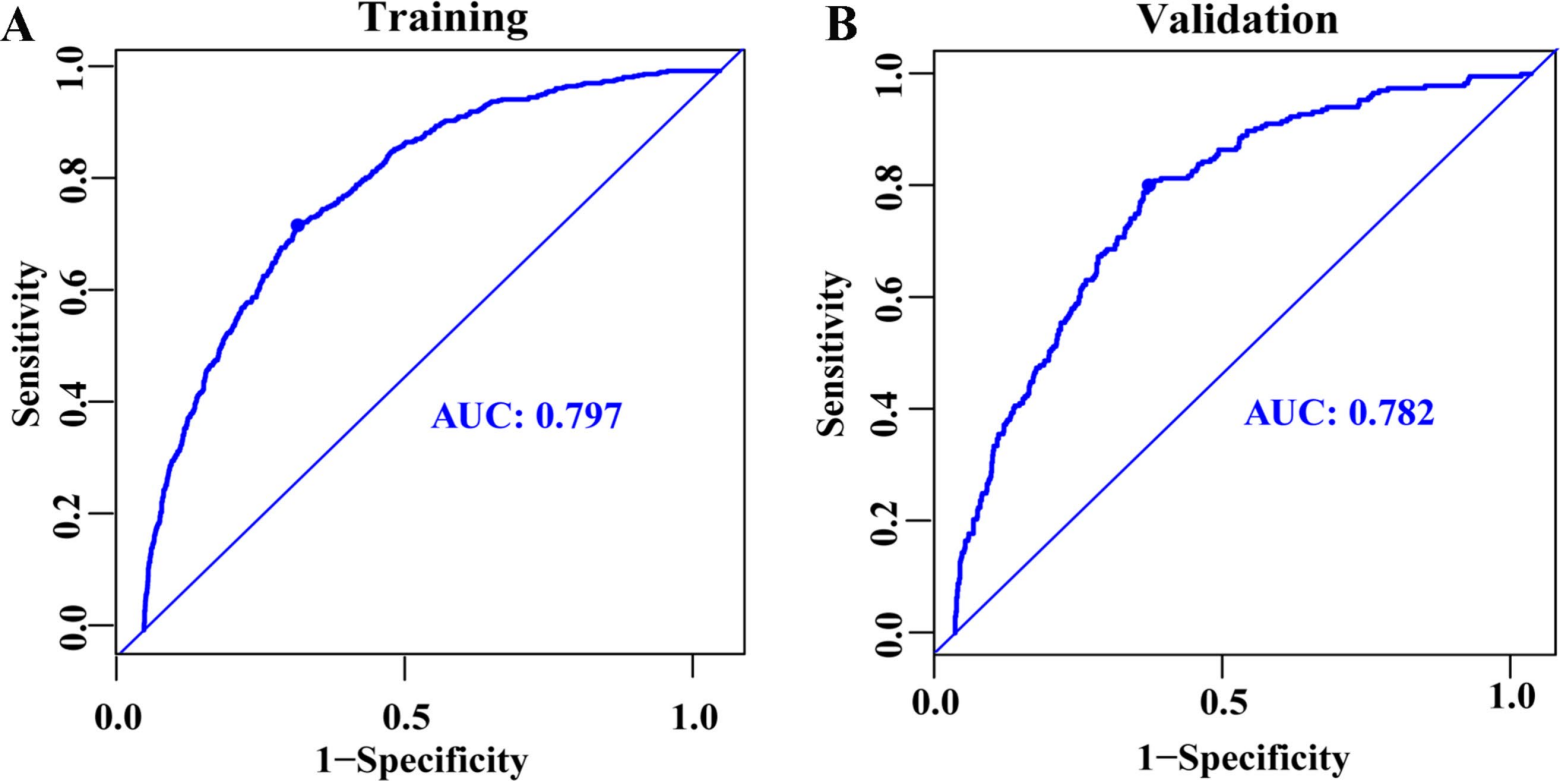
ROC curves for predicting frailty among middle-aged and older adults, presented for the training dataset **(A)** and the validation dataset **(B)**.

LASSO regression was employed to identify the relevant factors most associated with frailty. The LASSO regression model selected nine factors with non-zero coefficients in the training dataset, including the hs-CRP/HDL-C ratio, age, BMI, gender, FITPR, smoke, DM status, hypertension status, and stroke status (Figure 4) in the final model. These nine independent predictors were subsequently included in the nomogram construction (Figure 5). The ROC analysis demonstrated that the nomogram exhibited strong discriminatory power, with an AUC of 79.7% (95% CI 77.7–81.75%) in the training set and an AUC of 78.2% (95% CI 75.0–81.4%) in the validation set (Figure 6).

## 4 Discussion

The prevalence of frailty is increasing with the aging population. Frailty, characterized by functional decline and increased risk of adverse outcomes such as falls, hospitalizations, and complications (26), arises from multisystem dysregulation and metabolic imbalances (4). Its diagnosis remains clinically challenging, and delayed recognition may lead to increased healthcare costs. Systemic inflammation has been linked to frailty risk (18). A 3-year longitudinal study in China revealed that hs-CRP contributes to frailty development (10). Besides, higher HDL-C levels have been associated with improved well-being and physical health (15, 27). HDL-C, with its roles in reverse cholesterol transport, anti-inflammatory, and antioxidant functions, may exert protective effects against age-related diseases (28). The hs-CRP/HDL-C ratio, a composite marker of inflammation and lipid status, reflects the balance between pro- and anti-inflammatory processes. This ratio has been recognized as a clinically relevant biomarker for cardiovascular diseases(CVD), metabolic dysfunction-associated steatotic liver disease (MASLD), advanced liver fibrosis, and poor outcomes in acute ischemic stroke (16, 29, 30), due to its simplicity and clinical relevance. These conditions, in turn, are known contributors to frailty. CVD may promote frailty by reducing physical activity and functional capacity (31). Similarly, the progression of MASLD is frequently accompanied by sarcopenia, insulin resistance, and chronic inflammation, all of which are implicated in frailty development (32). In older stroke patients, diminished multi-organ function and physiological reserve may reduce the ability to respond to external stressors, thereby increasing frailty risk (33). Therefore, an elevated hs-CRP/HDL-C ratio may contribute to frailty development through its associations with chronic comorbidities and systemic dysfunction. In our nationally representative U.S. sample, we observed a significant and non-linear association between the hs-CRP/HDL-C ratio and frailty risk among middle-aged and older adults. Collectively, these findings suggest that the hs-CRP/HDL-C holds promise as a potential biomarker for frailty assessment in this population.

The precise mechanisms underlying the association between the hs-CRP/HDL-C ratio and frailty remain elusive. Albumin, a vital serum protein component, exerts nutritional and antioxidant functions, whereas globulin, an immunoglobulin type, is indicative of systemic inflammatory activity (34, 35). Hypoalbuminemia, which worsens with age, is associated with both malnutrion and inflammation (36), and prior studies have identified albumin as a potential biomarker for frailty (37, 38). In our analysis, the hs-CRP/HDL-C ratio was negatively associated with albumin and positively related to globulin. These plasma proteins partially mediated the relationship between the hs-CRP/HDL-C ratio and frailty, suggesting their roles in inflammation-related frailty pathogenesis.

Identifying critical predictors is essential for the prevention and early management of frailty. In this study, LASSO regression was employed for variable selection. Our analysis identified the hs-CRP/HDL-C ratio, age, BMI, gender, FITPR, smoke, DM status, hypertension status, and stroke status as key predictors of frailty. Frailty, a clinical syndrome characterized by diminished muscle mass and physical activity, is closely linked to aging (39). Sex differences also influence frailty risk; men generally have greater muscle mass than women due to higher androgen levels (40). Elevated BMI is linked to increased comorbidities, such as diabetes and cardiovascular disease, and has been related to higher frailty risk in older adults (41). Beyond physiological factors, social health factors such as poverty is associated with frailty by reflecting cumulative health risks over time (42). Besides, tobacco use has been associated with telomere shortening, sarcopenia, reduced gait speed, and physical inactivity, indicating its potential role in age-related frailty (43). Hypertension may elevate frailty risk through mechanisms involving polypharmacy, physical and cognitive decline, and fall susceptibility in older adults (44). Similarly, DM promotes frailty via dysglycemia, sarcopenia, decreased muscle strength, and multimorbidity (45). In older patients, stroke may contribute to frailty through neurological dysfunction, sarcopenia and malnutrition (46). Our study supports a positive association between the hs-CRP/HDL-C ratio and frailty. Based on the identified predictors, we constructed a nomogram to serve as a predictive tool for frailty risk. This model may assist clinicians to efficiently identify individuals at high risk of frailty and initiate appropriate interventions in a timely manner.

Several limitations were identified in our study. First, we did not evaluate the cumulative impact of the hs-CRP/HDL-C ratio on frailty status. Second, the retrospective nature of self-reported data, such as smoking and alcohol use, may lead to recall bias, potentially leading to misclassification. Third, due to the observational design, causal relationships between hs-CRP/HDL-C and frailty cannot be determined. Although we adjusted for a wide range of potential confounders, there may be residual confounding from unmeasured factors that influence the association. While NHANES provides a nationally representative sample, our findings may not be applicable to individuals with specific health conditions-such as rare or severe conditions. Therefore, future study in specific populations is needed to validate our results and improve clinical applicability. Moreover, multiple biomarkers reflecting oxidative stress, nutrition, and aging—such as oxidative balance score (OBS), composite dietary antioxidant index (CDAI), albumin, globulin, and klotho—have been proposed in relation to frailty (4). Previous studies showed that OBS or CDAI has significant associations with frailty (47, 48). Although our primary focus was the hs-CRP/HDL-C ratio, we also evaluated additional markers (e.g., albumin, globulin) and demonstrated their partial mediating roles in the association between hs-CRP/HDL-C ratio and frailty. Nonetheless, some relevant biomarkers were not included in our analysis, limiting our ability to capture the full biological complexity of frailty. Future prospective studies incorporating more comprehensive data, as well as longitudinal or interventional designs, are needed to validate these findings and further explore the underlying mechanisms.

## 5 Conclusion

In summary, this cross-sectional study of middle-aged and older participants demonstrated a positive association between frailty and the hs-CRP/HDL-C ratio. Plasma proteins, including albumin and globulin, were identified as mediators in this correlation. Through LASSO regression, the key factors correlated with frailty were identified, and a nomogram was subsequently developed to estimate frailty risk. These findings emphasize the critical role of the hs-CRP/HDL-C ratio in predicting frailty risk, highlighting its potential clinical utility.

## Data Availability

The datasets presented in this study can be found in online repositories: https://wwwn.cdc.gov/nchs/nhanes/. Further inquiries can be directed to the corresponding author.

## References

[1] Ofori-Asenso R Chin KL Mazidi M Zomer E Ilomaki J Zullo AR. Global incidence of frailty and prefrailty among community-dwelling older adults: a systematic review and meta-analysis. JAMA Netw Open. (2019) 2:e198398. doi: 10.1001/jamanetworkopen.2019.8398, PMID: 31373653 PMC6681553

[2] El Assar M Rodríguez-Sánchez I Álvarez-Bustos A Rodríguez-Mañas L. Biomarkers of frailty. Mol Asp Med. (2024) 97:101271. doi: 10.1016/j.mam.2024.101271 38631189

[3] Stenholm S Ferrucci L Vahtera J Hoogendijk EO Huisman M Pentti J. Natural course of frailty components in people who develop frailty syndrome: evidence from two cohort studies. J Gerontol A Biol Sci Med Sci. (2019) 74:667–74. doi: 10.1093/gerona/gly132, PMID: 30084927 PMC6477647

[4] Dzięgielewska-Gęsiak S Muc-Wierzgoń M. Inflammation and oxidative stress in frailty and metabolic syndromes-two sides of the same coin. Meta. (2023) 13:475. doi: 10.3390/metabo13040475, PMID: 37110134 PMC10144989

[5] Liu X Wang Y Shen L Sun Y Zeng B Zhu B. Association between frailty and chronic constipation and chronic diarrhea among American older adults: National Health and nutrition examination survey. BMC Geriatr. (2023) 23:745. doi: 10.1186/s12877-023-04438-4, PMID: 37968629 PMC10647084

[6] Yin MJ Xiong YZ Xu XJ Huang LF Zhang Y Wang XJ. Tfh cell subset biomarkers and inflammatory markers are associated with frailty status and frailty subtypes in the community-dwelling older population: a cross-sectional study. Aging. (2020) 12:2952–73. doi: 10.18632/aging.102789, PMID: 32039831 PMC7041730

[7] Westbrook R Chung T Lovett J Ward C Joca H Yang H. Kynurenines link chronic inflammation to functional decline and physical frailty. JCI Insight. (2020) 5:e136091. doi: 10.1172/jci.insight.136091, PMID: 32814718 PMC7455140

[8] Tuttle CSL Thang LAN Maier AB. Markers of inflammation and their association with muscle strength and mass: a systematic review and meta-analysis. Ageing Res Rev. (2020) 64:101185. doi: 10.1016/j.arr.2020.101185, PMID: 32992047

[9] Kamil RJ Betz J Powers BB Pratt S Kritchevsky S Ayonayon HN. Association of hearing impairment with incident frailty and falls in older adults. J Aging Health. (2016) 28:644–60. doi: 10.1177/0898264315608730, PMID: 26438083 PMC5644033

[10] Luo YF Cheng ZJ Wang YF Jiang XY Lei SF Deng FY. Unraveling the relationship between high-sensitivity C-reactive protein and frailty: evidence from longitudinal cohort study and genetic analysis. BMC Geriatr. (2024) 24:222. doi: 10.1186/s12877-024-04836-2, PMID: 38439017 PMC10913347

[11] Rolver MG Emanuelsson F Nordestgaard BG Benn M. Contributions of elevated CRP, hyperglycaemia, and type 2 diabetes to cardiovascular risk in the general population: observational and Mendelian randomization studies. Cardiovasc Diabetol. (2024) 23:165. doi: 10.1186/s12933-024-02207-0, PMID: 38730445 PMC11088022

[12] Chaturvedi S De Marchis GM. Inflammatory biomarkers and stroke subtype: an important new frontier. Neurology. (2024) 102:e208098. doi: 10.1212/wnl.0000000000208098, PMID: 38165352

[13] Milman S Atzmon G Crandall J Barzilai N. Phenotypes and genotypes of high density lipoprotein cholesterol in exceptional longevity. Curr Vasc Pharmacol. (2014) 12:690–7. doi: 10.2174/1570161111666131219101551, PMID: 24350928 PMC4087084

[14] Kosmas CE Sourlas A Guzman E Kostara CE. Environmental factors modifying HDL functionality. Curr Med Chem. (2022) 29:1687–701. doi: 10.2174/0929867328666210714155422 34269662

[15] Yuan Y Lin S Huang X Li N Zheng J Huang F. The identification and prediction of frailty based on Bayesian network analysis in a community-dwelling older population. BMC Geriatr. (2022) 22:847. doi: 10.1186/s12877-022-03520-7, PMID: 36368951 PMC9652858

[16] Gao Y Wang M Wang R Jiang J Hu Y Wang W. The predictive value of the hs-CRP/HDL-C ratio, an inflammation-lipid composite marker, for cardiovascular disease in middle-aged and elderly people: evidence from a large national cohort study. Lipids Health Dis. (2024) 23:66. doi: 10.1186/s12944-024-02055-7, PMID: 38429790 PMC10908181

[17] Hakeem FF Bernabé E Sabbah W. Association between oral health and frailty among American older adults. J Am Med Dir Assoc. (2021) 22:559–563.e2. doi: 10.1016/j.jamda.2020.07.023, PMID: 32859517

[18] Zhang H Liu X Wang X Jiang Y. Association of two novel systemic inflammatory biomarkers and frailty based on NHANES 2007-2018. Front Public Health. (2024) 12:1377408. doi: 10.3389/fpubh.2024.1377408, PMID: 38655524 PMC11036374

[19] Fan J Yu C Guo Y Bian Z Sun Z Yang L. Frailty index and all-cause and cause-specific mortality in Chinese adults: a prospective cohort study. Lancet Public Health. (2020) 5:e650–60. doi: 10.1016/s2468-2667(20)30113-4, PMID: 33271078 PMC7708389

[20] Guan Z Ma L Wu C. Association between serum klotho and physical frailty in middle-aged and older adults: finding from the National Health and nutrition examination survey. J Am Med Dir Assoc. (2023) 24:1173–1178.e2. doi: 10.1016/j.jamda.2023.02.103, PMID: 37001558

[21] Bao W Liu B Simonsen DW Lehmler HJ. Association between exposure to pyrethroid insecticides and risk of all-cause and cause-specific mortality in the general US adult population. JAMA Intern Med. (2020) 180:367–74. doi: 10.1001/jamainternmed.2019.6019, PMID: 31886824 PMC6990752

[22] Hupin D Roche F Gremeaux V Chatard JC Oriol M Gaspoz JM. Even a low-dose of moderate-to-vigorous physical activity reduces mortality by 22% in adults aged ≥60 years: a systematic review and meta-analysis. Br J Sports Med. (2015) 49:1262–7. doi: 10.1136/bjsports-2014-094306, PMID: 26238869

[23] Bao W Liu B Rong S Dai SY Trasande L Lehmler HJ. Association between bisphenol a exposure and risk of all-cause and cause-specific mortality in US adults. JAMA Netw Open. (2020) 3:e2011620. doi: 10.1001/jamanetworkopen.2020.11620, PMID: 32804211 PMC7431989

[24] Rattan P Penrice DD Ahn JC Ferrer A Patnaik M Shah VH. Inverse association of telomere length with liver disease and mortality in the US population. Hepatol Commun. (2022) 6:399–410. doi: 10.1002/hep4.1803, PMID: 34558851 PMC8793996

[25] Chen Z Song C Yao Z Sun J Liu W. Associations between albumin, globulin, albumin to globulin ratio and muscle mass in adults: results from the national health and nutrition examination survey 2011-2014. BMC Geriatr. (2022) 22:383. doi: 10.1186/s12877-022-03094-4, PMID: 35501822 PMC9059414

[26] Dent E Martin FC Bergman H Woo J Romero-Ortuno R Walston JD. Management of frailty: opportunities, challenges, and future directions. Lancet (London, England). (2019) 394:1376–86. doi: 10.1016/s0140-6736(19)31785-4, PMID: 31609229

[27] Wang S Liu M Yang S Wang J Jia W Cao W. Higher normal levels of triglyceride and low and high-density lipoprotein cholesterol might have a protective effect against activities of daily living disability within Chinese female centenarians: a cross-sectional, complete sample study. Clin Interv Aging. (2020) 15:225–37. doi: 10.2147/cia.S237505, PMID: 32110002 PMC7034296

[28] Asztalos BF Tani M Schaefer EJ. Metabolic and functional relevance of HDL subspecies. Curr Opin Lipidol. (2011) 22:176–85. doi: 10.1097/MOL.0b013e3283468061 21537175

[29] Liang B Qiu X Huang J Lu Y Shen H Ma J. Nonlinear associations of the hs-CRP/HDL-C index with metabolic dysfunction-associated steatotic liver disease and advanced liver fibrosis in US adults: insights from NHANES 2017-2018. Sci Rep. (2025) 15:4029. doi: 10.1038/s41598-025-88685-y, PMID: 39900651 PMC11791041

[30] Luwen H Lei X Qing-Rong O Linlin L Ming Y. Association between hs-CRP/HDL-C ratio and three-month unfavorable outcomes in patients with acute ischemic stroke: a second analysis based on a prospective cohort study. BMC Neurol. (2024) 24:418. doi: 10.1186/s12883-024-03929-0, PMID: 39468509 PMC11514845

[31] Takefuji Y. Exploring the connection between frailty and cardiovascular diseases. Arch Gerontol Geriatr. (2024) 124:105449. doi: 10.1016/j.archger.2024.105449, PMID: 38669728

[32] Karakousis ND Chrysavgis L Chatzigeorgiou A Papatheodoridis G Cholongitas E. Frailty in metabolic syndrome, focusing on nonalcoholic fatty liver disease. Ann Gastroenterol. (2022) 35:234–42. doi: 10.20524/aog.2022.0705, PMID: 35599934 PMC9062844

[33] HuangYNYanFHWangXYChenXLChongHYSuWL. Prevalence and risk factors of frailty in stroke patients: a meta-analysis and systematic review. J Nutr Health Aging. (2023) 27:96–102. doi: 10.1007/s12603-023-1879-z, PMID: 36806864

[34] Lai KJ Hsieh YP Chiu PF Lin PR. Association of albumin and globulin with mortality risk in incident peritoneal dialysis patients. Nutrients. (2022) 14:2850. doi: 10.3390/nu14142850, PMID: 35889807 PMC9324370

[35] Huang CY Liou SY Kuo WW Wu HC Chang YL Chen TS. Chemiluminescence analysis of antioxidant capacity for serum albumin isolated from healthy or uremic volunteers. Luminescence. (2016) 31:1474–8. doi: 10.1002/bio.3132, PMID: 27062681

[36] Zhang L Yang P Yin F Zhang J Zhao B Zhou J. Association between frailty and hypoproteinaemia in older patients: meta-analysis and systematic review. BMC Geriatr. (2024) 24:689. doi: 10.1186/s12877-024-05275-9, PMID: 39154175 PMC11329991

[37] Mailliez A Guilbaud A Puisieux F Dauchet L Boulanger É. Circulating biomarkers characterizing physical frailty: CRP, hemoglobin, albumin, 25OHD and free testosterone as best biomarkers. Results of a meta-analysis. Exp Gerontol. (2020) 139:111014. doi: 10.1016/j.exger.2020.111014, PMID: 32599147

[38] Yamamoto M Adachi H Enomoto M Fukami A Nakamura S Nohara Y. Lower albumin levels are associated with frailty measures, trace elements, and an inflammation marker in a cross-sectional study in Tanushimaru. Environ Health Prev Med. (2021) 26:25. doi: 10.1186/s12199-021-00946-0, PMID: 33607942 PMC7893938

[39] Cruz-Jentoft AJ Baeyens JP Bauer JM Boirie Y Cederholm T Landi F. Sarcopenia: European consensus on definition and diagnosis: report of the European working group on sarcopenia in older people. Age Ageing. (2010) 39:412–23. doi: 10.1093/ageing/afq034, PMID: 20392703 PMC2886201

[40] Conforto R Rizzo V Russo R Mazza E Maurotti S Pujia C. Advances in body composition and gender differences in susceptibility to frailty syndrome: role of osteosarcopenic obesity. Metab Clin Exp. (2024) 161:156052. doi: 10.1016/j.metabol.2024.156052, PMID: 39490438

[41] Sun Q Xia X He F. Longitudinal association between body mass index (BMI), BMI trajectories and the risk of frailty among older adults: a systematic review and meta-analysis of prospective cohort studies. Arch Gerontol Geriatr. (2024) 124:105467. doi: 10.1016/j.archger.2024.105467, PMID: 38728821

[42] Stolz E Mayerl H Waxenegger A Freidl W. Explaining the impact of poverty on old-age frailty in Europe: material, psychosocial and behavioural factors. Eur J Pub Health. (2017) 27:1003–9. doi: 10.1093/eurpub/ckx079, PMID: 29020312 PMC5881693

[43] Park S Kim SG Lee S Kim Y Cho S Kim K. Causal linkage of tobacco smoking with ageing: Mendelian randomization analysis towards telomere attrition and sarcopenia. J Cachexia Sarcopenia Muscle. (2023) 14:955–63. doi: 10.1002/jcsm.13174, PMID: 36696951 PMC10067476

[44] Shi J Tao Y Chen S Zhou Z Meng L Duan C. Interaction between hypertension and frailty and their impact on death risk in older adults: a follow-up study. BMC Geriatr. (2024) 24:187. doi: 10.1186/s12877-024-04793-w, PMID: 38402390 PMC10893602

[45] Chi CY Wang J Lee SY Chao CT Hung KY Chien KL. The impact of glucose-lowering strategy on the risk of increasing frailty severity among 49,519 patients with diabetes mellitus: a longitudinal cohort study. Aging Dis. (2023) 14:1917–26. doi: 10.14336/ad.2023.0225, PMID: 37196125 PMC10529743

[46] Wei J Wang J Chen J Yang K Liu N. Stroke and frailty index: a two-sample Mendelian randomisation study. Aging Clin Exp Res. (2024) 36:114. doi: 10.1007/s40520-024-02777-9, PMID: 38775917 PMC11111486

[47] Liu Y Han Y Gao Y Yao N Wang Y Wang F. The association between oxidative balance score and frailty in adults across a wide age spectrum: NHANES 2007-2018. Food Funct. (2024) 15:5041–9. doi: 10.1039/d4fo00870g, PMID: 38651948

[48] Wu Y Cheng S Lei S Li D Li Z Guo Y. The association between the composite dietary antioxidant index and frailty symptoms: mediating effects of oxidative stress. Clin Interv Aging. (2024) 19:163–73. doi: 10.2147/cia.S448354, PMID: 38332967 PMC10849906

