# Supplementary material for: Cross-sectional association between hs-CRP/HDL-C ratio and physical frailty among middle-aged and older adults: findings from a population-based study
Source: Front Public Health. 2025 May 9;13:1564206. doi: 10.3389/fpubh.2025.1564206 (PMC12098052; doi:10.3389/fpubh.2025.1564206)
Supplement: Supplementary file 1 [file Table_1.docx]

**Supplementary Table S1 List of 49 health deficits included in the frailty index.**

| **Cognition** | **Depressive Symptoms** | 35.doctor told you have diabetes |
| --- | --- | --- |
| 1.Experience confusion/memory problems | 18.have little interest in doing things | 36.ever told you had weak/failing kidneys |
| **Dependence** | 19.feeling down, depressed, or hopeless | 37.urine leakage bother you? |
| 2.Managing money difficulty | 20.trouble sleeping or sleeping too much | **Hospital Utilization and Access to Care** |
| 3.walking for a quarter mile difficulty | 21.feeling tired or having little energy | 38.general health condition |
| 4.walking up ten steps difficulty | 22.poor appetite or overeating | 39.health now compared with 1 year ago |
| 5.stooping, crouching, kneeling difficulty | 23.feeling bad about yourself | 40.overnight hospital patient in last year |
| 6.lifting or carrying difficulty | 24.trouble concentrating on things | 41.times receive healthcare over past year |
| 7.house chore difficulty | **Comorbidities** | 42.number of prescription medicines taken |
| 8.preparing meals difficulty | 25.doctor ever said you had arthritis | **Physical Performance and Anthropometry** |
| 9.standing up from armless chair difficulty | 26.ever told you had thyroid problem | 43.body mass index (kg/m^2) |
| 10.getting in and out of bed difficulty | 27.ever told you had chronic bronchitis | **Laboratory Values** |
| 11.using fork, knife, drinking from cup difficulty | 28.ever told you had cancer or malignancy | 44.glycohemoglobin(%) |
| 12.dressing yourself difficulty | 29.ever told had congestive heart failure | 45.red blood cell count (million cells/ul) |
| 13.standing for long periods difficulty | 30.ever told you had coronary heart disease | 46.hemoglobin (g/dl) |
| 14.grasp/holding small objects difficulty | 31.ever told you had angina/angina pectoris | 47.red cell distribution width (%) |
| 15.attending social event difficulty | 32.ever told you had heart attack | 48.lymphocyte percent (%) |
| 16.leisure activity at home difficulty | 33.ever told you had a stroke | 49.segmented neutrophils percent (%) |
| 17.push or pull large objects difficulty | 34.ever told you had high blood pressure |  |
